# Orthodontic apps at fingertips

**DOI:** 10.1186/s40510-014-0036-y

**Published:** 2014-05-30

**Authors:** Mayuresh Jagannath Baheti, Nandlal Toshniwal

**Affiliations:** Department of Orthodontics and Dentofacial Orthopedics, Rural Dental College, Loni, 413736 Maharashtra India

**Keywords:** Smartphone, Applications, Orthodontics

## Abstract

**Background:**

Smartphone usage has spread to many settings including that of healthcare and dentistry with numerous potential and realized benefits. The ability to download custom-built software applications (apps) has created new opportunities for orthodontists to integrate technology into clinical practice and patients to collect the information about orthodontics and help them during their treatment. The purpose of this study is to provide a summary of the orthodontic apps currently available for orthodontic patients as well as ‘practicing clinicians’.

**Method:**

Three smartphones and two tablets were used to search three operating systems (Android, Apple, and Windows) using the keywords ‘braces’, ‘orthodontist’, ‘model analysis’, and ‘orthodontics’.

**Results:**

Android and Apple operating systems accumulate all of the apps that are thought to be related to orthodontic clinicians and patients. Clinician's apps (17) are those related to orthodontic news (2), publication (4), products (3), and diagnosis (4) and practice management (3) while patient apps (17) are those related to orthodontic education (4), simulator (5), related to reminding patients about elastic wear (3), progress tracker of treatment (4), and orthodontic products (1).

**Conclusion:**

In the generation of technology, the use of smartphones and tablets has made life simple. The use of these technologies can be a boon both for the orthodontist and the patients as it aids both in treatment planning and progress in enhancing the treatment outcome.

**Electronic supplementary material:**

The online version of this article (doi:10.1186/s40510-014-0036-y) contains supplementary material, which is available to authorized users.

## Background

The latest generations of smartphones are increasingly viewed as handheld computers rather than as phones, due to their powerful onboard computing capability, capacious memories, large screens, and open operating systems that encourage application development [1]. Smartphones are now providing routine access to information in ways that were previously not possible, and this includes the area of medical education.

Once upon a time, phones were used exclusively for conversing with other people *via* telephone calls and text messages. They have a number of per-loaded software programs. But, the development of smartphones has created new opportunities to integrate mobile technology into daily clinical practice. A smartphone is a cellular telephone with an integrated computer that is capable of performing a broad array of tasks, including running various downloadable applications (apps) that typically are not associated with a cellular phone. The existence of smartphones can be traced as early as 1992 [2], then in 1993, IBM launched the Simon, a touch screen phone with integrated email, fax, calendar, and notepad; it was not until nearly 10 years later development of the Palm and Blackberry in 2001 and 2002, respectively, that consumers began to use mobile devices capable of wireless information services and web browsing. Release of the iPhone in 2007 included features not found on previous devices and led the way for developers to create a library of apps available to consumers [2].

An app is typically a small specialized program downloaded on to a mobile device [3]. An app is accessed using a smartphone that connects, *via* an internet portal, to a library of apps. The users can browse the library and search for specific apps that serve their needs [4]. Owing to their portability, ability to update, speed, and simplicity, smartphone apps are an ideal tool for quick reference or when accessing a desktop computer would not be feasible [5]. As a result, smartphones can serve as quick reference tools for train enquiry, flight enquiry, ticket booking, daily expense monitoring, check their bank balance, etc.

Despite the widespread prevalence of smartphones and apps available for general utilities, even there are some apps specifically designed for medical and dental field [6]. As medical and dental apps have been one of the fastest growing categories of programs and include various programs designed specifically for orthodontics. For orthodontics, there is now a wealth of information available, much of which can be freely accessed by our patients.

With smartphone use becoming more widespread, the medical community has embraced this technology with a number of apps already available to patients assisting them in smoking cessation [7] and for pain management [8] as two examples. Applications for clinicians in orthopedic surgery [2], ophthalmology [9, 10], and radiology [11] have already been reported as well as apps for students in medical education [6].

This article provides a summary of the orthodontic apps currently available for orthodontic patients as well as practicing clinicians.

## Method

Three smartphones and two tablets were used to access their respective app stores till 4th December 2013. A Samsung galaxy S4 with android OS v 4.2.2 (Jelly Bean) and a Samsung Galaxy tab 2 10.1 with android OS v 4.0 (Ice Cream sandwich) were used to search the Google play store. An iPhone 4 with iOS 6 and Apple ipad Mini MD528LL with iOS 6 were used to search Apple App Store. Finally, A Nokia Lumia 800 with OS windows phone 7.5 was used to search the windows phone marketplace.

Search was carried out using four keywords: ‘braces’, ‘orthodontist’, ‘model analysis’, and ‘orthodontics’. The play store includes a brief description of the apps that has been provided by the developer. Information of each app was recorded including the cost of the app and brief information about the app.

Apps that featured lite and full versions were also counted. Non-health, general dental, and medical apps were excluded. Only apps directly relating to the field of orthodontics were examined.

## Results

The results of the search, with the aid of the three operating systems, using the four keywords were so many apps in which some are related to entertainment and utility; dental apps included those marketing dental practices, guides for dental assisting, and even dental game. Apps in the above were excluded and orthodontic-related apps were divided into two main categories: (1) apps for orthodontic clinicians and (2) apps for orthodontic patients. These two categories were further divided into different categories according to their ability of function. The total number of apps for all search words together and directly relevant to orthodontics are shown in Table 1. No orthodontic apps were found for windows phone. Apps related to orthodontic clinicians and patients are summarized in Tables 2 and 3, respectively.

**Table 1:** **Orthodontically relevant app**

OS	Category	Number
Android	For clinician	12
	For patient	10
	Practice marketing	23
	Simulator	4
	Total	49
Apple	For clinician	15
	For patient	13
	Practice marketing	30
	Simulator	12
	Total	70

**Table 2:** **Apps relevant to orthodontic clinician**

Apps for clinician	Operating system	Cost	Description
Orthotown	iPhone	0	New and active topics and cases of the day
	Android		
Orthodontic Exam Pro	Android	2,000.00/-Rs	For those who want face exams and take exams
American Journal of Orthodontics & Dentofacial Orthopedics	iPad	0	American Journal of Orthodontics and Dentofacial Orthopedics
Dental Press	Apple	0	Dental Press Orthodontics and Facial Orthopedics Journal
Wired Orthodontics	Apple	0	Orthodontic laboratory and manufacturer app
Orthodontic Products	Apple	0	Orthodontic product news and techniques magazine
SimplyCeph	iPad	44,900/-Rs	Cephalometric analyzer
SmileCeph International	iPad	27,900/-Rs	Cephalometric analyzer
iModel Analysis	Android	0	Orthodontic model analyzer
Bolton Calc	Android	68.99/-Rs	Tooth-width ratio analyzer
ShareSmilez	Apple	0	Integrates with your social media with patients

**Table 3:** **Apps relevant to orthodontic patients**

Apps for patients	Operating system	Cost	Description
Learn about Braces	Android	0	Educates the patient about malocclusion and how braces can benefit them
Straighten Me	Apple	0	Information on how to treat an orthodontic emergency
iBrace Help	Apple	0	Information on braces, their care and wear
Invisible Braces Scan	Android	0	Checks whether patient is suitable for invisible braces
	Apple		
Alignermeter	Android	0	Checks whether patient is suitable for invisible braces
	Apple		
Brace Accelerator	iPhone	55/-Rs, 54.37/-Rs	Reminds patient to wear and change their elastics
	Android		
Rubberband Reminder	iPhone	110/-Rs, 109.84/-Rs	Reminds patient to wear and change their elastics
	Android		
WAMBCO Orthodontic Treatment	Apple	0	Tracks treatment progress
	Android		
The Invisible Orthodontist	Apple	0	Tracks treatment progress for invisible braces
3M Incognito	Apple	0	Information on the Incognito system

### Apps for orthodontic clinicians

Out of 15 apps considered to be designed for orthodontic clinicians, two are related to orthodontic news. Through the *Orthotown* app, we can post new topics, respond to threads, check out the active topics and cases of the day and access Orthotown magazine. The *Orthodontic exam pro* app is the perfect app for those who want face exams worldwide or take up P.G. Exams like MS Ortho or MOrth RCS. Four apps were used to access publications. The app *American Journal of Orthodontics & Dentofacial Orthopedics, AJO-DO* provides access to AJO-DO, a monthly clinical journal published by AJO-DO. The app *Dental press* provides access to the Journal of Orthodontics and Maxillary Orthopedics with the objective to publish not only scientific studies, but also other text of interest for clinical orthodontics. The app *Intl Asso for Orthodontics* provides access to the International Journal of Orthodontics, a quarterly peer- reviewed clinical journal. The *Orthodontic Products* app is a print and digital publication covering topics such as efficient treatment, staff management, and marketing methods.

Three apps for orthodontic products were found. *Dentaurum Dental Products* app offers the current Dentaurum Group catalogues for the field of orthodontics and other fields. *Wired Orthodontics* is produced by Wired Orthodontics Ltd. for the information and reference. *Carriere Ortho 3D* app provides animation of the Carriere Distaliser and assists in patient education about Carriere products.

Four apps were related to diagnosis. *SimplyCeph* allows you to maintain patient records with cephalometric analysis, photographic analysis, and 3D model analysis. *SmileCeph International* app only does cephalometric analysis. *iModel Analysis* performs several model analysis such as Bolton analysis, Howes analysis, Pont and Linder-Harth analyses, etc. *Bolton Calc* app allows you to enter the dimensions of teeth and then calculates if tooth-size discrepancy exists. Two practice management apps were also found; *Dental Appoint Manager* app allows you to save information of the patient, easily book appointment, and reminds to send mail to patient. *ShareSmilez* app integrates with your social media accounts and makes it easy for patients to become your fan during their office visit.

### Apps for orthodontic patients

Four orthodontic education apps for patients were found. *Learn about Braces* app educates the patient about malocclusion and the condition that can cause some discomfort and pain and one can see how braces can benefit them. *Straighten Me* uses decision trees which take users to the most appropriate slideshow video with information on how to treat their orthodontic emergency. *iBrace Help* is an informative app for people wearing braces or those who are interested in getting orthodontic work done. *Straighten Me* and *iBrace Help* apps are available for iPhones only. Five simulator apps were available. Out of these, three required the user to pay a charge to download the app. The two other apps that do not charge for download are *Invisible Braces Scan* app which lets you take a scan of your teeth to check whether the patient is suitable for invisible braces and *Alignermeter* app which determines if a patient is a candidate for invisible braces in a quick and easy process.

**Figure 1:**
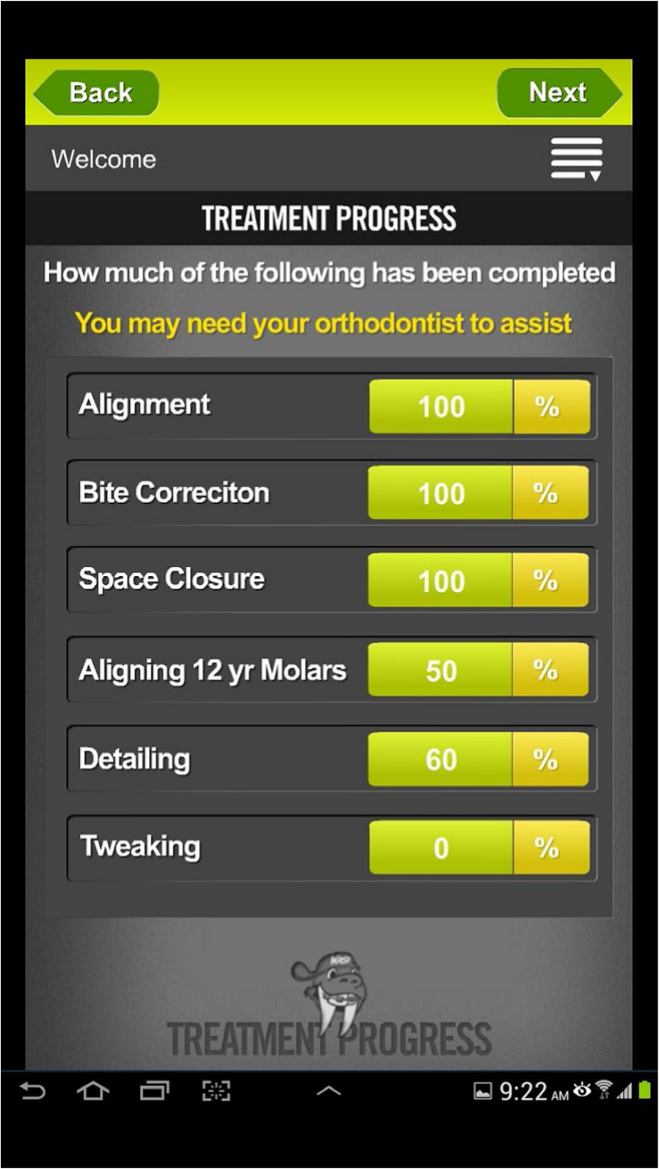
**Example of WAMBCO Orthodontic Treatment app: treatment progress.**

**Figure 2:**
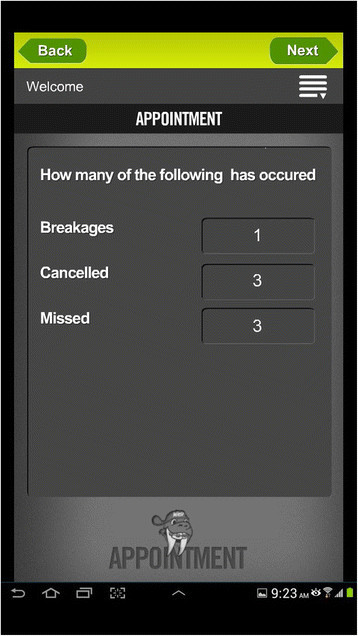
**Example of WAMBCO Orthodontic Treatment app: appointment.**

Three of the patient apps were reminders. *Brace Accelerator* and *Rubberband Reminder* send alerts that remind patients to wear their orthodontic elastics. But, both are paid apps. *Align On Time* app allows patient and clinicians to generate aligner change schedules. Four apps were found for patient progress tracking of treatment. Out of these, one is a paid app. *WAMBCO Orthodontic Treatment* app is developed by Orthodontist. In this app, the patient will need to enter some of information based on their treatment, to estimate how long the process should take. The patient will probably also need the assistance of their orthodontist to estimate how much of the treatment has been completed (Figures 1 and 2). The *Invisible Orthodontist* app assists patients with their progress through their treatment plan for invisible braces. *SorrisoOrtho* is the first app created with the sole objective to enhance the orthodontic experience. The main feature of this app is the ability to customize the app for each orthodontic office. *3M Incognito* was the only one app found targeting patients about their appliance system. This was the app found for iPhone only.

## Discussion

Searches for orthodontic apps yield results only for Apple iPhone, iPad, Android smartphone, and tab. This is perhaps a reflection of market impact of these two operating systems.

It was considered that the four keywords ‘braces’, ‘orthodontist’, ‘model analysis’, and ‘orthodontics’ should source most of the orthodontic apps. However, this keyword search gives a large proportion of apps not related to those orthodontic apps. This applied 100% for the Windows apps. Android and Apple operating systems produce 49 and 70 orthodontic related apps, respectively.

A number of apps were found to be useful for orthodontic clinicians and could be downloaded without cost. The orthodontic journals might allow access to their publications *via* apps, particularly as online journals become more prevalent. Apps to assist in performing diagnosis may also be useful, both for those in orthodontic training and for practicing clinicians. These apps are those which assist in performing cephalometry and calculating tooth-size discrepancy. Apps linked to commercial companies such as Wired Orthodontics and Carrier Ortho 3D give information related to their company products. Some apps related to practice management gives a clinician paperless work in this paper-free world in an easy way to manage patients efficiently.

The apps considered suitable for patients regarding orthodontic education gives information about malocclusion and the condition regarding it and how braces can benefit them. The apps suitable for patients utilizing alerts for elastic and aligner wear could be useful for our patients to complete treatment early. Orthodontic treatment time can be reduced and managed better if patients are able to access information on appliance care and treatment progress.

Since apps are often small specialized programs, the depth of information available to the clinician and patient can often be limited. Clinician apps such as those for tracing lateral cephalogram (SimplyCeph), calculating tooth size discrepancies (iModel analysis), and staying in touch with patients (Sharesmilez) do as their titles suggest.

Similarly, patient apps were often limited to few functions. Rubberband Reminder is a sophisticated alarm reminder for changing the rubber and Align On Time reminds patients to change their aligners. WAMBCO Orthodontic Treatment app is developed by Orthodontist and it keeps the treatment progress and tells the patient how much time is still required.

It should be considered that by the time of publication, some apps will have been added, while others will have been removed. The further study can be done to know how many clinicians and patients access smartphones and are willing to download orthodontic apps.

## Conclusion

In the generation of technology, the use of smartphones and tablets has made life simple. The use of these technologies can be a boon both for the orthodontist and the patients as it aids both in treatment planning and progress in enhancing the treatment outcome.

## Authors’ original submitted files for images

Below are the links to the authors’ original submitted files for images. Authors’ original file for figure 1 Authors’ original file for figure 2
