# Supplementary figures and images for: Orthodontic apps at fingertips
Source: Prog Orthod. 2014 May 30;15:36. doi: 10.1186/s40510-014-0036-y (PMC4883981; doi:10.1186/s40510-014-0036-y)

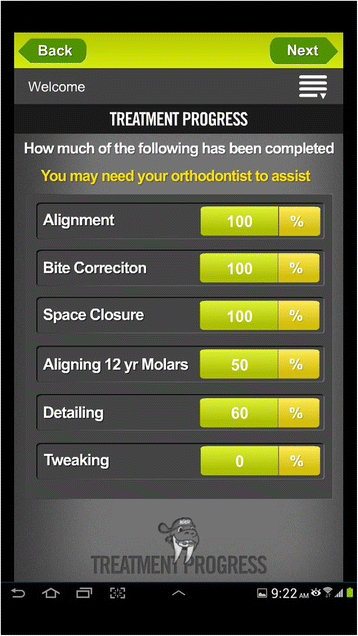

Supplement: Supplementary file 1 — Authors’ original file for figure 1 [file 40510_2014_36_MOESM1_ESM.gif]

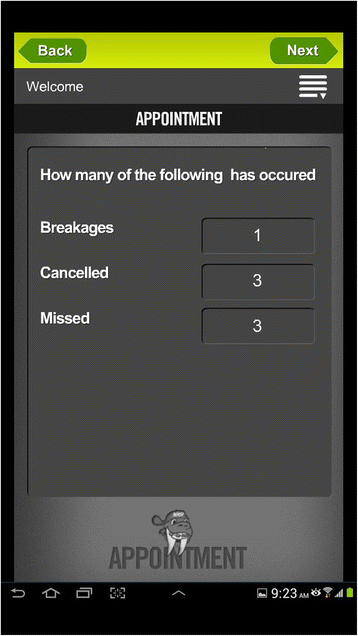

Supplement: Supplementary file 2 — Authors’ original file for figure 2 [file 40510_2014_36_MOESM2_ESM.gif]
